# Who is this person and what did she accomplish?

**DOI:** 10.3201/eid3204.251083

**Published:** 2026-04

**Keywords:** sanitation, women in medicine, women’s health, Civil War, England, United States

Here is a clue: This 19th-century physician organized women nurses during the US Civil War and promoted sanitary reforms when disease killed more soldiers than combat.

Who is she?

**A)** Dorothea Dix **B)** Lucy Stone **C)** Clara Barton **D)** Florence Nightingale **E)** Elizabeth Blackwell

Decide first, then see next page for the answer.
