# Supplementary material for: Who is this person and what did she accomplish?
Source: Emerg Infect Dis. 2026 Apr;32(4):653–6. doi: 10.3201/eid3204.251083 (PMC13094825; doi:10.3201/eid3204.251083)
Supplement: Related texts

# Other: Elizabeth Blackwell, 19th Century Pioneer of Public Health

**Authors:** Nicolas Hoffmann

**Affiliations:** Marist School, Atlanta, Georgia, USA; Georgia Gwinnett College, Lawrenceville, Georgia, USA

This is a photograph of Elizabeth Blackwell (1821–1910), an Anglo-American physician who helped open the medical profession to women in the United States (Figure). Born in Bristol, England, she emigrated with her family to New York, New York, USA, in 1832 and later settled in Cincinnati, Ohio, where financial hardship after her father’s death led her and her sisters to support themselves through teaching. Raised in an abolitionist and reform-minded household, she resolved to pursue medicine despite widespread opposition.

**Figure:** Elizabeth Blackwell, seated in profile (ca. 1850–60). Source: Harvard University, Schlesinger Library on the History of Women in America, Radcliffe Institute.

Blackwell was rejected by numerous medical schools because she was a woman before Geneva Medical College admitted her in 1847, after the faculty submitted her application to the all-male student body for a vote. The students, reportedly believing the proposal to be a jest, voted unanimously to accept her. She graduated in 1849, becoming the first woman in the United States to earn a medical degree. She furthered her study in Paris and London, including work under James Paget, who first described Paget’s disease. Blackwell treated an infant with neonatal conjunctivitis and accidentally contracted the infection in her left eye, leaving her partially blind and ending her hopes of becoming a surgeon but she became committed to sanitation. She returned to New York in 1851 to establish private practice.

Blackwell’s early years in practice were difficult. Female medical practitioners of the time were often assumed to be abortionists, but Blackwell opposed both abortion and artificial contraception. She published *The Laws of Life with Special Reference to the Physical Education of Girls* in 1852, the first book on the subject written by a woman, and lectured widely on women’s health and hygiene. In 1857, she founded the New York Infirmary for Indigent Women and Children, which became a model for female-led healthcare and medical education. The infirmary offered clinical training for women in medicine and later was among the first American women’s medical colleges. Throughout that time, Blackwell wrote widely, submitting to journals but, more important, evangelizing the cause of women in medical care; she wrote *Medicine as a Profession for Women*, a textbook for the program she was starting.

However, when the Civil War began in 1861, the nation mobilized for the war effort. The onslaught of sick and wounded soldiers overwhelmed Union and Confederate military and civilian medical systems at the outset of the war. Both armies rapidly expanded their forces, but the infrastructure to support the wounded—especially medical supplies and trained personnel—lagged substantially. At the time, no nursing corps existed. The military and even most doctors did not consider nursing a profession. In hospitals, most nursing was done by strong men whose primary job was to move patients or to hold them down for surgery, which was performed without anesthetic. In the military, that job typically fell to enlisted men of low rank, who received a modest additional wage for their service.

The scale and severity of the conflict quickly exposed the inadequacy of that model. Disease, malnutrition, and grievous battlefield injuries demanded more competent and organized medical care. In the Union Armies alone, according to *The Medical and Surgical History of the War of the Rebellion (1861–65)*, published by the US Surgeon General’s Office, 20,587,450 transmittable diseases and 879,717 nontransmittable conditions led to the deaths of 142,522 soldiers off the battlefields. Furthermore, there were >60,000 amputations. In response, a nationwide push began, especially in the North, to train and recruit professional nurses.

One of the most substantial of those efforts came from the Blackwell clinic, which became a central hub for nursing training. Hundreds of women received structured medical instruction there, and the school produced nurses widely seen as more effective and capable than their untrained counterparts. Members of New York’s congressional delegation urged Congress to appoint Blackwell to lead the Union nursing effort, citing her leadership and medical expertise. Despite Blackwell’s qualifications and long-standing abolitionist commitments—shaped in part by her association with reformers such as William Lloyd Garrison—Congress instead selected Dorothea Dix. Lawmakers viewed Dix, whose national reputation stemmed from her asylum and prison reform campaigns, as the more politically acceptable choice.

Congress appointed Dix as Superintendent of Army Nurses, making her the first woman to head a federal bureau. Blackwell continued her work independently, expanding nursing training through the Women’s Central Association of Relief, an outgrowth of the New York Infirmary for Women and Children.

Tensions emerged between the 2 reformers. Dix imposed strict age restrictions, 35–50 years, and appearance standards for nurses. She sought “matronly persons of experience… and serious disposition” and rejected applicants she deemed too young or insufficiently matronly. Blackwell, in contrast, emphasized medical training and professional competence. Dix’s rigid administrative style drew criticism from military officials and reformers alike; General George McClellan reportedly nicknamed her “Dragon Dix,” and Jane Grey Swisshelm called her a “self-sealing can of horror, tied up in red tape.” Dix’s style provoked criticism from fellow reformers, including Blackwell.

Although not a founder of the US Sanitary Commission (USSC), Blackwell supported its medically oriented and reform-driven approach to wartime care. The USSC emphasized sanitary inspection, improved hospital organization, and professional standards in nursing, principles aligned with Blackwell’s advocacy for trained female medical workers. However, tensions soon emerged between Dix and other reformers over control of nurse appointments and qualifications. War Department General Order No. 351 in 1863 reduced Dix’s unilateral authority, allowing surgeons and military officials greater discretion in staffing decisions and opening space for more formally trained women to serve. Throughout the war, Blackwell continued organizing women physicians and promoting professional medical education. The broader sanitary movement, including the efforts of the USSC, the US Christian Commission, and independent volunteers such as Clara Barton, helped shift American medicine toward greater institutional organization and professionalization in the postwar era.

Throughout her life, Elizabeth Blackwell championed abolition, public health reform, sanitation, and the professional training of women physicians. Although not an outspoken suffragist, she collaborated with leading advocates for women’s rights and helped expand opportunities for women in medicine and science. Her enduring legacy lies not only in the institutions she founded but in the generations of women physicians she trained and inspired. Blackwell died on May 31, 1910, at her home in Hastings, England, remembered as a pioneer who overcame systemic exclusion to reshape both American and British medicine.

## References

[1] Blackwell E. The laws of life with special reference to the physical education of girls. New York: Putnam; 1852.

[2] Blackwell E. Medicine as a profession for women. New York: Trustees of the New York Infirmary for Women; 1860.

[3] Devine S. Learning from the wounded: the Civil War and the rise of American medical science. Chapel Hill (NC): The University of North Carolina Press; 2014.

[4] Dix D. Circular, no. 8. Washington: US Government Printing Office; 1862.

[5] Faust DG. This republic of suffering: death and the American Civil War [reprint]. New York: Vintage; 2009.

[6] Maxwell WQ. Lincoln’s fifth wheel: the political history of the U.S. Sanitary Commission. New York: Longmans, Green & Co.; 1956.

[7] Nimura JP. The Doctors Blackwell: how two pioneering sisters brought medicine to women - and women to medicine. New York: W.W. Norton & Company; 2021.

[8] American Battlefield Trust. Dorothea Lynde Dix [cited 2025 Jul 17]. https://www.battlefields.org/learn/biographies/dorothea-lynde-dix

[9] US Government Publishing Office. The medical and surgical history of the War of Rebellion (1861–65). Washington: The Office; 1870.
